# Combining neoadjuvant chemotherapy with PD-1/PD-L1 inhibitors for locally advanced, resectable gastric or gastroesophageal junction adenocarcinoma: A systematic review and meta-analysis

**DOI:** 10.3389/fonc.2023.1103320

**Published:** 2023-01-26

**Authors:** Zhen Yuan, Hao Cui, Shuyuan Wang, Wenquan Liang, Bo Cao, Liqiang Song, Guibin Liu, Jun Huang, Lin Chen, Bo Wei

**Affiliations:** ^1^ School of Medicine, Nankai University, Tianjin, China; ^2^ Department of General Surgery, The First Medical Center, Chinese PLA General Hospital, Beijing, China; ^3^ Department of Radiotherapy, The Fifth Medical Center, Chinese PLA General Hospital, Beijing, China

**Keywords:** gastric adenocarcinoma, gastroesophageal junction adenocarcinoma, PD-1/PD-L1 inhibitors, immune checkpoint inhibitors, neoadjuvant immunotherapy

## Abstract

**Background:**

Immune checkpoint inhibitors (ICIs) have shown promising prospects in locally advanced, resectable gastric or gastroesophageal junction adenocarcinoma (GC/GEJC) immunotherapy, but their efficacy in neoadjuvant settings remains unclear. This study aimed to assess the efficacy and safety of integrating programmed cell death 1 (PD-1)/programmed cell death ligand 1 (PD-L1) inhibitors into neoadjuvant chemotherapy (NACT) of GC/GEJC treatment.

**Methods:**

PubMed, Cochrane Library, Embase, ClinicalTrials.gov, and main oncology conference databases were systematically searched up to 19 November 2022, and randomized controlled trials (RCTs) and cohort studies that evaluated the efficacy and safety of PD-1/PD-L1 inhibitors plus NACT were included. The main outcomes were pathological complete response (pCR), major pathological response (MPR), R0 resection rate, and treatment-related adverse events (TRAEs).

**Results:**

A total of 753 patients from 20 prospective studies were included in this meta-analysis. The pooled pCR and MPR rates from studies reporting were 21.7% [95% confidence interval (CI), 18.1%–25.5%] and 44.0% (95% CI, 34.1%–53.8%), respectively. The pooled incidence rate of total TRAEs was 89.1% (95% CI, 82.7%–94.3%), and the incidence rate of grade 3 to 4 TRAEs was 34.4% (95% CI, 17.8%–66.5%). The pooled R0 resection rate was reported to be 98.9% (95% CI, 97.0%–99.9%). Subgroup analysis has not found significant differences in efficacy and safety among different PD-1/PD-L1 inhibitors. Moreover, the efficacy in patients with positive PD-L1 expression (combined positive score ≥1) was comparable with that in the entire study population [pCR, 22.5% vs. 21.2% (p > 0.05); MPR, 48.6% vs. 43.7% (p > 0.05)].

**Conclusion:**

This systematic review and meta-analysis found that PD-1/PD-L1 inhibitors combined with NACT for locally advanced GC/GEJC were well tolerated and may confer therapeutic advantages. The integration of ICIs into NACT has shown the potential for application in any PD-L1 expression population.

## 1 Introduction

Gastric cancer (GC) is one of the most common malignant tumors, which ranks fifth of the incidence rate and fourth of the mortality, with more than 1 million estimated new cases annually (1). East Asia is a region with a high incidence rate of GC. Unlike a higher proportion of early GC in Japan and South Korea, about 80% of patients suffer from advanced GC (AGC) due to inappropriate dietary habits and low prevalence of early cancer screening, which cause a serious health burden on life in China (2, 3). At present, many studies have proved that the combination of gastrectomy and perioperative therapy like chemotherapy, targeted therapy, or immunotherapy can prolong the survival and improve the quality of life of patients with AGC, bringing prospects and opportunities for the investigation of optimized treatment (4, 5).

*Helicobacter pylori* infection is the most well-described risk factor for GC. Other risk factors include male sex, older age, smoking, alcohol consumption, high salt intake, low vegetables and fruits intake, and low socioeconomic status (6). New molecular classifications, such as The Cancer Genome Atlas classification, divide GC into four subtypes: EBV, MSI, CIN, and GS, which could better reflect tumor characteristics and show prognostic values than that based on morphological or histopathological features (7). Alteration of the tumor microenvironment (TME) has also been widely reported in GC with upregulated programmed cell death ligand 1 (PD-L1) expression observed, which correlated with tumor immune escape (8). As GC is a highly heterogeneous malignant disease and the therapeutic effect is still unsatisfactory, it is of great significance to elucidate the pathogenesis and explore better treatment means for GC.

In recent years, as the representative of immunotherapy, programmed cell death 1 (PD-1)/PD-L1 inhibitors have become a superior option for treating AGC. The mechanism of action is briefly through selective blockade of the immune checkpoints of the T-cell surface to activate T cells for effective anti-tumor response (9). Most perspectives considered that the combination of immunotherapy and chemotherapy could synergistically enhance the anti-cancer effect. This might be due to the increased recognition and elimination of the tumor by the host immune system with the combination of drugs, as well as reduced immunosuppression of the TME and enhanced antitumor efficacy (10, 11). Some authoritative randomized controlled trials (RCTs) like Checkmate-649 (12) and Attraction-4 (13) also proved that the combination of PD-1/PD-L1 inhibitors and chemotherapy in unresectable AGC has significantly improved disease control rate and brought long-term survival benefits compared with chemotherapy alone. This high-level evidence makes chemotherapy combined with immunotherapy become the first-line treatment modality for unresectable AGC (14).

Since the MAGIC study (15) established the predominance of neoadjuvant chemotherapy (NACT) in the treatment of locally AGC (LAGC), this preoperative treatment modality has been widely recognized for its satisfied therapeutic effect on reducing tumor stage, inhibiting tumor micrometastasis, increasing R0 resection rate, and even achieving pathological complete response (pCR) and acquiring long-term survival benefits (16). The important value of immunotherapy in AGC draws surgeons’ attention to applying it in the neoadjuvant treatment of LAGC. Teng et al. (17) first reported the clinical use of NACT and immunotherapy for patients with LAGC, and the results of this phase II single-arm study demonstrated that patients who accepted sintilimab in combination with CapeOX regimen acquired 97.2% R0 resection rate and 19.4% pCR rate, which seemed superior to the results of neoadjuvant CapeOX regimen only in the previous studies (18, 19). In the last 2 years, an increasing number of prospective studies have started to focus on the effect of NACT combined with immunotherapy and obtained initial results. Therefore, we summarized the current literatures and conducted this meta-analysis in an attempt to comprehensively analyze the therapeutic safety and clinicopathological evaluation of NACT combined with immunotherapy for LAGC and to provide a reference for surgeons to rationally select the effective treatment modalities.

## 2 Methods

### 2.1 Literature search strategy

The systematic literature search was conducted in PubMed, Cochrane Library, Embase, ClinicalTrials.gov, and several main oncology conference databases, including American Society of Clinical Oncology (ASCO) Meeting, European Society for Medical Oncology (ESMO) Meeting, and American Association for Cancer Research (AACR) Meeting up to 19 November 2022. Search terms using the medical subject headings (MeSH) were as follows: (“immunotherapy” [MeSH] OR “immune checkpoint inhibitors” [MeSH] OR “nivolumab” OR “pembrolizumab” OR “camrelizumab” OR “sintilimab” OR “toripalimab” OR “tislelizumab” OR “atezolizumab” OR “durvalumab” OR “avelumab”) AND (“neoadjuvant therapy” [MeSH] OR “perioperative period” [MeSH]) AND (“esophagogastric junction” [MeSH] OR “stomach neoplasm” [MeSH] OR “gastroesophageal cancer” OR “gastroesophageal junction cancer”). We also searched unpublished studies and ongoing clinical trials of neoadjuvant immunochemotherapy in locally advanced GC/GEJC. All studies were limited to English language and human subjects.

### 2.2 Inclusion criteria

Inclusion criteria according to the PICOS principle was listed as follows: patients: patients with newly diagnosed resectable locally advanced (T1N1-3M0 or T2-3NanyM0) GC/GEJC; intervention: administrated with NACT combined with PD-1/PD-L1 inhibitors; and outcomes: efficacy and security indicators, including pathological complete response (pCR), major pathological response (MPR), R0 resection rate, treatment-related adverse events (TRAEs), and grade 3 to 4 TRAEs. pCR and MPR are both graded according to Becker criteria of Tumor Regression Grade (TRG). Studies design: RCTs, non-RCTs, and prospective cohort studies.

### 2.3 Data extraction

Two authors (HC and ZY) independently carried out the literature selected and data extraction, and disagreements were resolved by discussing with the third author (BW). The following information was extracted: name of clinical trials, name of first author, number of clinical trials, publication year, region, tumor site, neoadjuvant treatment regimen, type of PD-1/PD-L1 inhibitors, study phase, study design, number of patients, main inclusion criteria, main outcome indicators, median age, proportion of males, resection rate, R0 resection rate, pCR rate, MPR rate, incidence of TRAEs, incidence of grade 3 to 4 TRAEs, incidence of serious adverse events (SAE), PD-L1 expression level (detected before neoadjuvant therapy), and mismatch repair (MMR) status.

### 2.4 Quality assessment and risk of bias

The Cochrane ROBINS-I tool was applied to assess the quality and bias of included studies, which incorporating assessment for bias in the domains of selection, attrition, detection, performance, and reporting (**Supplementary Figure 1**). Two reviewers (JH and LS) estimated the quality of studies independently, and disagreements were discussed with the third investigator. Publication bias was evaluated by Begg’s funnel plots and Egger’s test.

### 2.5 Statistical analysis

The meta-analysis was carried out using R software (version 4.2.1). Because most of studies were single-arm trails, five transformation approaches (PRAW, PAS, PFT, PLN, and PLOGIT) were conducted, and the approach by which the lowest heterogeneity was achieved was adopted for further analysis. Moreover, the heterogeneity among studies was evaluated by the Cochrane’s Q test and I^2^ statistical; if the heterogeneity was negligible (I^2^ < 50%, p > 0.1), the fixed-effects model was adopted; and if the heterogeneity was significant (I^2^ > 50%, p < 0.1), the random-effects model was adopted. A p-value < 0.05 was considered statistically significant.

## 3 Results

**Figure 1:**
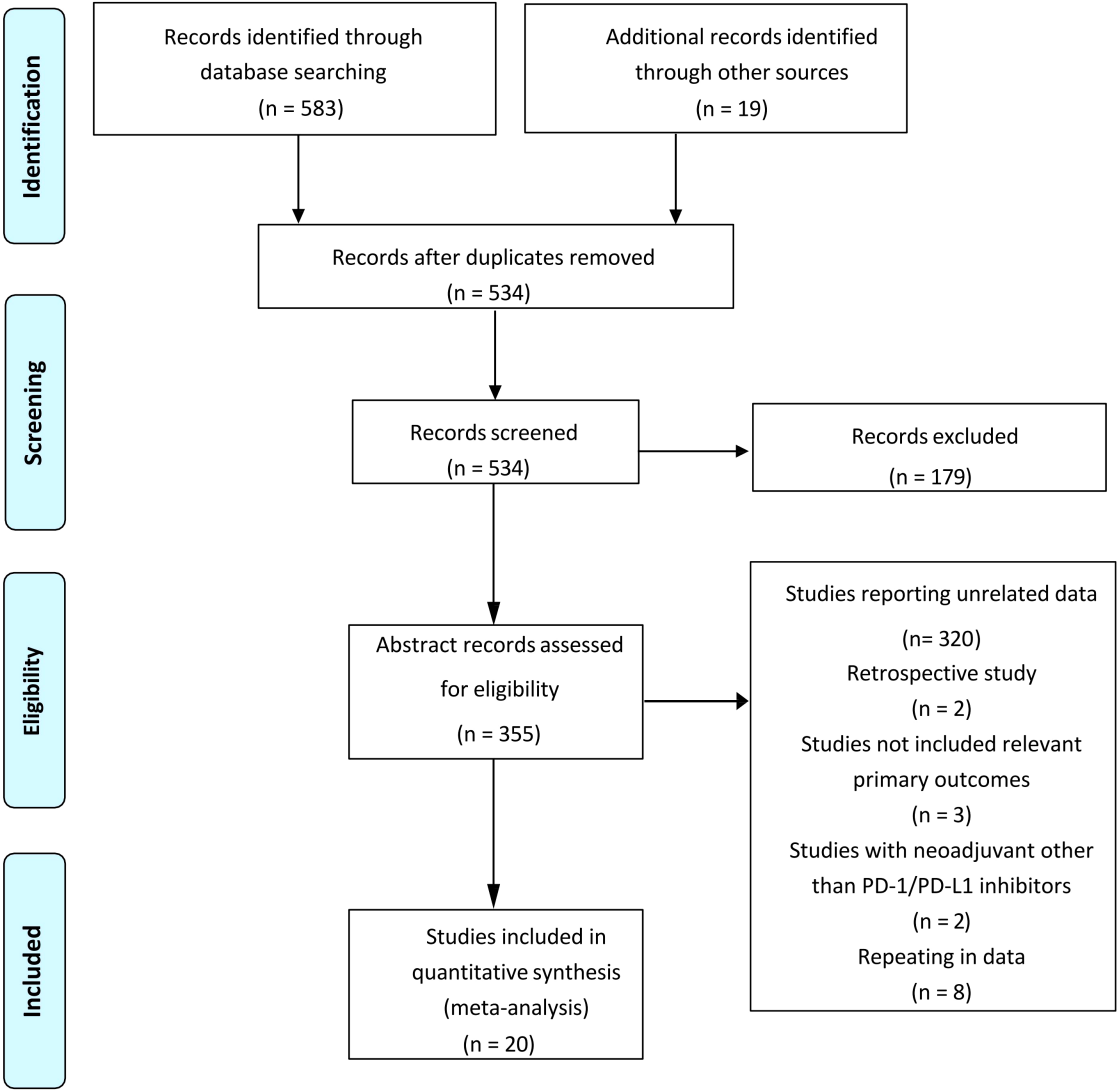
Flowchart depicting the search strategy.

The original search identified a total of 602 publications, and the study retrieval process is depicted in **Figure 1**. Twenty studies met the inclusion criteria, including a total number of 753 patients, were included in this meta-analysis (17, 20–38). Of these, 15 studies reported that patients received neoadjuvant immunotherapy combined with chemotherapy, which our meta-analysis mainly focused on. Another five studies reported patients who received neoadjuvant immunochemotherapy with radio therapy or anti-VEGF drugs. The characteristics of all studies are listed in **Table 1**. Publication bias was estimated by Begg’s funnel plot and Egger’s test and showed overall limited, as illustrated in **Supplementary Figure 2**.

**Table 1:** Main characteristics of included studies.

Source	Year	NCT number	Country	Neoadjuvant treatment regimen	Phase	Study design	No. of patients	Main inclusion criteria	Median age, years	Proportion of males (%)
H. Jiang et al. (39)	2022	NCT04065282	China	3 cycles of sintilimab + CapeOX	II	Single-center, single-arm	36	cT3-4NanyM0 GC/GEJC	65.5 (43–76)	24 (66.7)
C. Xu et al. (20)	2022	–	China	SOX + atezolizumab + apatinib	–	Two-center, cohort study	30	LAGC	–	–
DANTE (21)	2022	NCT03421288	Germany and Switzerland	8 cycles of atezolizumab + 4 cycles of FLOT	IIb	Randomized, multicenter, two-arm	146	cT ≥2 and/or N + GC/GEJC	–	–
W. Sun et al. (22)	2022	NCT03488667	USA	3 cycles of pembrolizumab + 4 cycles of mFOLFOX6	II	Single-center, single-arm	37	T1N1-3M0 EC/GC/GEJC	–	30 (81.1)
J. Tang et al. (23)	2022	NCT04755543	China	3 cycles DDP + 5-FU + LP002	Ib	Open-label, single-arm	30	PD-L1 positive, cT2-4a, Nany, M0 GC/GEJC	64.5 (50–74)	26 (86.7)
PANDA, L. Verschoor et al. (24)	2022	NCT03448835	Netherlands	A single cycle of atezolizumab + 4 cycles of atezolizumab + DOC	II	Single-arm, single center	20	Resectable GC/GEJC	62 (47–77)	18 (90.0)
X. Ding et al. (25)	2022	ChiCTR2100043572	China	3 cycles of sintilimab + SOX	II	Single-arm, single center	21	Clinical stage II-IV GC/GEJC	56 (31–72)	10 (47.6)
K. Tao et al. (26)	2022	NCT04890392	China	3 cycles of tislelizumab + SOX.	II	Single-arm, open-label, non-randomized	32	Local advanced G/GEJC	–	–
H. Guo et al. (35)	2022	ChiCTR2000030414	China	4 cycles of sintilimab + CapeOX	II	Single-arm, open-label	30	cT3-4 N + M0 GC	62 (30–72)	18 (60.0)
M. Zhu et al. (37)	2022	–	USA	Pembrolizumab + CROSS	Ib/II	Single-center, single-arm	31	cT1–3NanyM0 GEJC	–	–
Z. Liu et al. (32)	2022	ChiCTR2000030610	China	4 cycles of FLOT + camrelizumab	–	Single-center, randomized, controlled clinical study	51	Locally advanced GC/GEJC	63 (28–72)	36 (70.6)
Neo-Capture, Z. Jiang et al. (33)	2022	NCT04119622	China	4 cycles of toripalimab + CapeOX	II	Single-center, single-arm	31	cT3-4 Nany M0 or T1-2 N2-3 M0 GC/GEJC	61 (34–72)	21 (80.0)
S. Li et al. (29)	2021	NCT03878472	China	2 cycles of camrelizumab + apatinib + S-1 ± oxaliplatin	II	Single-center, single-arm	25	cT4a/bN + GC	63 (48–70)	19 (76.0)
Y. Liu et al. (38)	2021	NCT03939962	China	4 cycles of carrelizumab + mFOLFOX6	II	Single-center, single arm	49	stage ≥ T2 GC/GEJC	57 (29–72)	43 (72.0)
SHARED, W Jia et al. (28)	2021	ChiCTR1900024428	China	4 cycles of sintilimab + cCRT	II	Multicenter, single-arm	28	III-IVA GC/GEJC	67 (47–81)	24 (85.7)
Neo-PLANET, Z. Tang et al. (30)	2021	NCT03631615	China	Chemoradiotherapy + CapeOX + 5 cycles of carrelizumab	II	Single-center, single arm	36	cT3-4aN + M0 GC	65.5 (35–72)	28 (77.8)
A.G. Raufi et al. (31)	2021	NCT02918162	USA	3 cycles of CapeOX + pembrolizumab	II	Multicenter, single-arm	36	Locally advanced GC/GEJC	65	–
Gastrimm-001, H. Li et al. (34)	2021	NCT04354662	China	4 cycles of FLOT + toripalimab	II	Single-center, single-arm	36	cT≥2 or cN + GC/GEJC	–	–
T. Alcindor et al. (36)	2020	NCT03288350	Canada	4 cycles of avelumab + mDCF	II	Single-center, single-arm	28	cT3 and/or cN + GC/GEJC	–	–
N. Li et al. (27)	2020	NCT04341857	China	4 cycles of FLOT + 3 cycles of sindilimab	II	Single-arm, open-label	20	T3/N + or higher stage GC/GEJC	–	–

GC, gastric adenocarcinoma; GEJC, gastroesophageal junction adenocarcinoma; EC, esophageal adenocarcinoma; ICI, immune checkpoint inhibitor.

### 3.1 Evaluation of efficacy outcomes

The efficacy- and safety-related outcomes were extracted from eligible studies and illustrated in **Table 2**. The data of pCR rate, MPR rate, and R0 resection rate were available in 14, 12, and 14 studies, respectively. The pooled rates of pCR and MPR from trials using neoadjuvant immunotherapy plus chemotherapy were 21.7% [95% confidence interval (CI), 18.1%–25.5%] and 44.0% (95% CI, 34.1%–53.8%), respectively. The pooled R0 resection rate was 98.9% (95% CI, 97.0%–99.9%). Only patients who received neoadjuvant immunotherapy combined with chemotherapy were included in the pooled analysis, and the results of the addition of neoadjuvant radiotherapy or anti-VEGF drug, apatinib, were analysis separately.

**Table 2:** Research data on outcomes reported in the studies.

Source	Treatment mode	ICI	No. of patients	Patients with resection	R0 resection rate (n, %)	pCR rate (n, %)	MPR rate (n, %)	Incidence of TRAEs (n, %)	Incidence of ≥3 TRAEs (n, %)
H. Jiang	ICI + chemo	Sintilimab	36	36	35 (97.2)	7 (19.4)	17 (47.2)	33 (91.7)	10 (27.8)
DANTE study	ICI + chemo	Atezolizumab	146	146	135 (92.5)	35 (24.0)	71 (48.6)	–	130 (89.0)
W. Sun	ICI + chemo	Pembrolizumab	37	27	27 (100.0)	5 (18.5)	–	–	21 (77.8)
J. Tang	ICI + chemo	LP002	30	27	24 (88.9)	1 (3.7)	3 (11.1)	–	–
PANDA study	ICI + chemo	Atezolizumab	20	20	20 (100.0)	9 (45.0)	14 (70.0)	–	–
X. Ding	ICI + chemo	Sintilimab	21	21	21 (100.0)	7 (33.3)	8 (38.1)	–	2 (9.5)
K. Tao	ICI + chemo	Tislelizumab	32	30	30 (100.0)	8 (26.7)	17 (56.7)	24 (80.0)	4 (13.3)
N. Li	ICI + chemo	Sintilimab	20	16	15 (93.8)	3 (18.8)	10 (62.5)	–	–
Y. Liu	ICI + chemo	Camrelizumab	49	45	42 (93.3)	4 (8.9)	10 (22.2)	–	–
A.G. Raufi	ICI + chemo	Pembrolizumab	36	29	29 (100.0)	7 (24.1)	13 (44.8)	–	18 (62.1)
Z. Liu	ICI + chemo	Camrelizumab	26	26	26 (100.0)	3 (11.5)	7 (26.9)	–	–
Neo-Capture study	ICI + chemo	Toripalimab	31	27	26 (96.3)	3 (11.1)	4 (14.8)	–	4 (14.8)
Gastrimm-001 study	ICI + chemo	Toripalimab	36	28	28 (100.0)	7 (25.0)	12 (42.9)	26 (94.0)	–
H. Guo	ICI + chemo	Sintilimab	30	30	30 (100.0)	10 (33.3)	19 (63.3)	27 (90.0)	–
M. Zhu	ICI + chemo + radio	Pembrolizumab	31	29	28 (96.6)	7 (24.1)	–	–	–
T.Alcindor	ICI + chemo	Avelumab	28	27	26 (96.3)	6 (22.2)	–	–	–
S. Li	ICI + chemo + apatinib	Camrelizumab	25	18	18 (100.0)	3 (16.7)	5 (27.8)	–	–
C. Xu	ICI + chemo + aptinib	Atezolizumab	30	28	26 (92.9)	2 (7.1)	6 (21.4)	–	–
SHARED study	ICI + chemo + radio	Sintilimab	28	19	19 (100.0)	8 (42.1)	14 (73.7)	–	11 (57.9)
Neo-PLANET study	ICI + chemo + radio	Camrelizumab	36	36	33 (91.7)	12 (36.4)	16 (48.5)	36 (100.0)	28 (77.8)

pCR, pathological complete response; MPR, major pathological response; TRAEs, treatment-related adverse events; ICI, immune checkpoint inhibitor.

**Figure 2:**
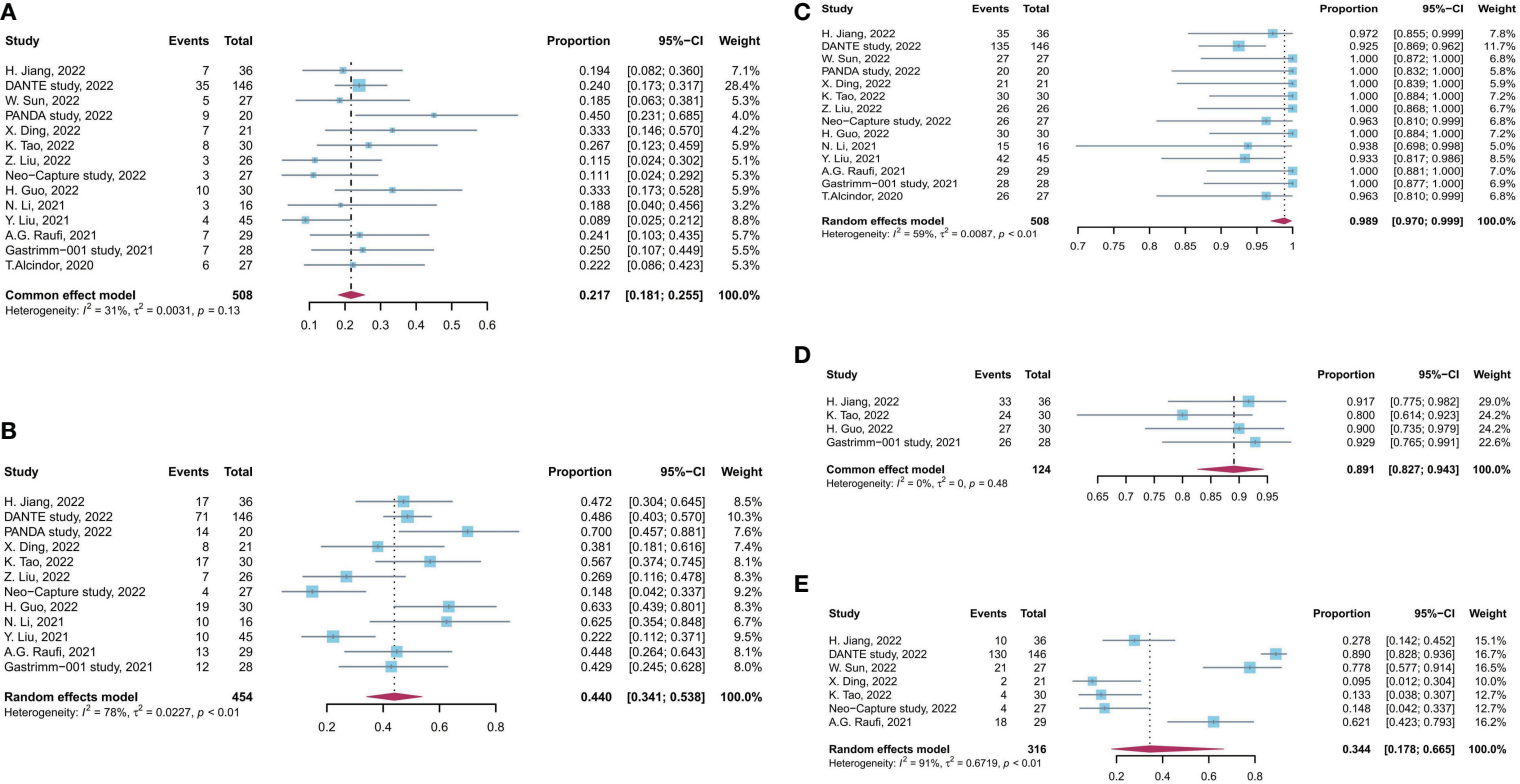
Forest plot of the efficacy and safety evaluation of neoadjuvant immunochemotherapy for locally advanced GC/GEJC. **(A)** pCR rate; **(B)** MPR rate; **(C)** R0 resection rate; **(D)** incidence of TRAEs; **(E)** incidence of grade 3 to 4 TRAEs. pCR, complete pathological response; MPR, major pathological response; TRAEs, treatment-related adverse event; GC, gastric adenocarcinoma; GEJC, gastroesophageal junction adenocarcinoma.

No significant heterogeneity was observed among results of pCR rates (I^2^ = 31%, p = 0.13, **Figure 2**), and fixed-effects model was adopted. Meanwhile, a high level of heterogeneity was observed for MPR (I^2^ = 78%, p < 0.01) and R0 resection rate (I^2^ = 59%, p < 0.01). Sensitivity analysis did not find the source of heterogeneity of MPR rates, whereas, after sensitivity analysis and DANTE study were excluded, the heterogeneity of R0 resection rate was decreased (**Supplementary Figure 3**).

### 3.2 Safety of neoadjuvant immunotherapy

The incidence rate of TRAEs was used to assess the safety of neoadjuvant immunotherapy combined with chemotherapy. The pooled incidence of TRAEs was 89.1% (95% CI, 82.7%–94.3%), based on four studies with available data. The pooled incidence rate of grade 3 to 4 TRAEs was 34.4% (95% CI, 17.8%–66.5%), supported by seven studies with available data (I^2^ = 31%, p = 0.13, **Figure 2**). There is a significant heterogeneity for grade 3 to 4 TRAEs (I^2^ = 91%, p < 0.01), and sensitivity analysis did not find the source of heterogeneity (**Supplementary Figure 3**). No significant heterogeneity was observed among results of TRAEs.

### 3.3 Subgroup analysis

**Figure 3:**
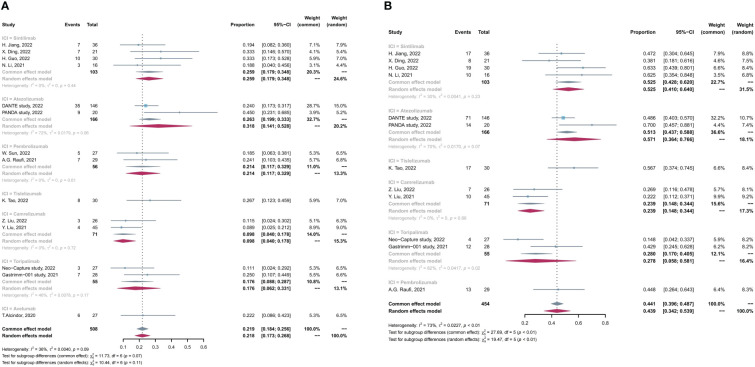
Subgroup analysis based on PD-1/PD-L1 inhibitors for **(A)** pCR rate and **(B)** MPR rate. pCR, complete pathological response rate; MPR, major pathological response rate.

Subgroup analysis was conducted on the basis of PD-1/PD-L1 inhibitor types and PD-L1 expression levels separately. Subgroup analysis has not found significant differences in efficacy and safety among different PD-1/PD-L1 inhibitors (**Figure 3**). Patients who received sintilimab- or atezolizumab-based neoadjuvant immunochemotherapy had a relatively better MPR rate compared with those who received camrelizumab- or toripalimab-based treatment (total, p < 0.01). For the incidence of grade 3 to 4 TRAEs, patients treated with sintilimab, tisleizumab, and toripalimab had a relatively lower incidence than those treated with atezolizumab or pembrolizumab (total, p < 0.01; **Supplementary Figure 4**), which were consistent with results in pairwise comparisons. No difference in R0 resection rate, pCR rate, or TRAE rate was found in subgroup analysis by PD-1/PD-L1 inhibitor types (**Supplementary Figure 4**).

**Figure 4:**
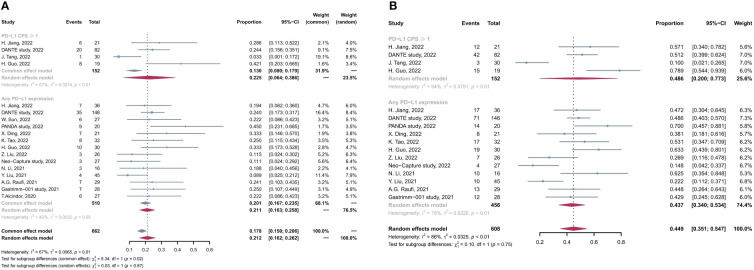
Subgroup analysis based on PD-L1 expression levels for **(A)** pCR rate and **(B)** MPR rate. pCR, complete pathological response rate; MPR, major pathological response rate.

Subsequently, we conducted subgroup analysis on the basis of PD-L1 expression levels, and four studies reported the data of efficacy in positive PD-L1 expression [defined as combined positive score (CPS) ≥1] subgroup. As shown in **Figure 4**, the pCR and MPR rates in patients with positive PD-L1 was comparable with those in the entire included patients with any PD-L1 expression levels (pCR, 22.5% in positive PD-L1 vs. 21.2% in any PD-L1, p > 0.05; MPR 48.6% in positive PD-L1 vs. 43.7% in any PD-L1, p > 0.05).

Furthermore, we additionally analyzed the efficacy and safety of neoadjuvant immunochemotherapy plus radiotherapy or anti-VEGF drug (apatinib) in locally advanced GC/GEJC (**Supplementary Figure 5**). In pairwise comparisons, compared with neoadjuvant immunochemotherapy only, neoadjuvant immunochemotherapy plus radiotherapy improved MPR rate (p = 0.01), but did not improve pCR rate (p > 0.05), and correlated with a higher incidence of grade 3 to 4 TRAEs (p < 0.01). Moreover, neoadjuvant immunochemotherapy plus apatinib was correlated with worse MPR rate than neoadjuvant immunochemotherapy only (p < 0.01) or neoadjuvant immunochemotherapy plus radiotherapy (p < 0.01).

## 4 Discussion

The combination of NACT and immunotherapy brings infinite possibility for elevating therapeutic effects for patients with locally advanced GC/GEJC. In this systematic review and meta-analysis, we summarized the efficacy and safety data of medication in the latest prospective studies to figure out the potential value for further application.

The major feature of neoadjuvant therapy was the possibility of tumor downstaging, which might achieve MPR or even pCR for sensitive individuals. These pathologically favorable responses have been shown to benefit patients in terms of long-term overall survival (OS) and disease-free survival (DFS) (40, 41). Therefore, pCR and MPR could be used as key indicators in the selection of optimal regimens. Previous studies have demonstrated that the pCR rate and MPR rate for patients with LAGC who received NACT was mostly 5.6%–16% (16, 42, 43) and 21.2%–27.6% (44, 45). In the present study, we found that the pooled pCR rate is 21.7% and that the pooled MPR rate is 44.0% in patients with LAGC who received NACT combined with immunotherapy, which seemed superior to that in patients who received NACT alone in previous studies. Currently, three studies have reported encouraging results that NACT combined with immunotherapy performed better in short-term outcomes than single NACT in LAGC treatment (**Supplementary Table 1**), and six relevant clinical trials are going (**Supplementary Table 2**). For further research, it is necessary to combine multi-omics techniques to predict the sensitive populations (46–48) and to discover clinical predictors associated with better pathologic responses (49), so that the therapeutic effect of LAGC will ultimately improve.

TRAE is widely used clinical indicators to evaluate the safety of perioperative treatment, and it is common in immunotherapy (50). Chemotherapy combined with immunotherapy has become the first-line treatment in AGC. The ATTRACTION-4 study in Asian population showed a comparable safety profile in the nivolumab-chemotherapy group compared with that in the placebo-chemotherapy group, with neutropenia being the most common grade 3 to 4 adverse event (13). The ORIENT-16 study in the Chinese population found that the incidence rate of grade ≥3 TRAEs in the sintilimab-chemotherapy group was comparable with that in the chemotherapy group (59.8% vs. 52.5%, p = 0.063) (51). A meta-analysis comparing the safety of different combinations of immunotherapy and chemotherapy regimens showed that the incidence rate of TRAE ≥3 grade was similar between PD-1 inhibitor with oxaliplatin-based chemotherapy and that with cisplatin-based chemotherapy (RR = 0.86, 95% CI, 0.66–1.12), whereas, among patients with oxaliplatin-based chemotherapy, the incidence rate of TRAE ≥3 times was comparable between those combining nivolumab and those combining sintilimab (52). When immunotherapy was applied in neoadjuvant therapy, a meta-analysis found a higher incidence of TRAEs in neoadjuvant ICI plus chemotherapy compared with neoadjuvant immunotherapy, in which the most common all-grade TRAEs were neutropenia, nausea, and alopecia; high-grade TRAEs were neutropenia, anemia, and aspartate aminotransferase and aspartate aminotransferase (AST) elevation; moreover, the low rates of treatment-related surgical delays and deaths further confirm the safety and feasibility of neoadjuvant immunotherapy (53). In present study, we found that the overall TRAE rate was 89.1%, and the incidence rate of grade 3 to 4 TRAEs was 34.4% in patients treated with NACT plus immunotherapy. With regard to dosing and perioperative mortality, four deaths among patients with atezolizumab combining FLOT (4/141, 3%) were reported by DANTE study (21). Moreover, Raufi et al. (54) reported that two of the three cases with grade 5 TRAEs among patients treated with pembrolizumab plus neoadjuvant CapeOX might attributable to treatment; no deaths were observed in the remaining patients during the dosing and perioperative period.

Furthermore, a significant heterogeneity was observed in grade 3 to 4 TRAEs in different ICI treatments (**Figure 2E**). Atezolizumab and pembrolizumab were correlated with a relatively high grade 3 to 4 TRAE rate, whereas the rate was relatively low in sintilimab, tislelizumab, and toripalimab. Detailed information of grade 3 to 4 TRAEs has been listed in **Supplementary Table 3**, and, partially, the difference can be attributed to different type of ICIs. Apart from the type of PD-1/PD-L1 inhibitors, different chemotherapy regimens may influence the effectiveness of the perioperative treatment (55). There have been controversial regarding the differences in terms of efficacy and safety for different regimen of NACT. Studies by Taieb et al. and Sah et al. reported that no statistically difference was observed in effectiveness between FLOT regimen and SOX regimen (56, 57). However, Grizzi et al. found that patients administrated with FLOT had better OS and DFS than those administrated with SOX (58). Studies included in this meta-analysis incorporate several NACT regimens, including SOX, CapeOX, FLOT, mFOLFOX6, and DOC. We have performed additional subgroup analysis by different chemotherapy regimens and illustrated that SOX performed better in pCR rate and MPR rate and correlated with lower incidence of grade 3 to 4 TRAEs than other regimens (**Supplementary Figure 6**). Moreover, different combination of ICI and chemotherapy regimen may affect the effectiveness of treatment, but the limited number of studies could not support relevant analysis, which need further investigation to make individualized treatment regimen for patients.

PD-L1 expression levels was considered to be associated with the effectiveness of PD-1/PD-L1 inhibitors (59); however, whether individuals with low PD-L1 expression could benefit from immunotherapy remains controversial. A study by Zhao et al. reported that patients with PD-L1 low expression could not benefit from ICI treatment (60). However, the Checkmate-649 and ORIENT-16 studies have illustrated that all-treated patients with advanced GC/GEJC had significant better OS and PFS, no matter what the expression levels of PD-L1 are (12). These findings also supported by the recent studies that the combining of ICIs and chemotherapy could improve the prognosis of patients with GC/GEJC (61, 62). Our results were consistent with the findings above. There are eight trials included that reported the PD-L1 status, and patients with positive PD-L1 status (CPS ≥ 1) had a comparable pCR and MPR compared with all-treated patients, which supported those who are administrated with PD-1/PD-L1 inhibitors regardless of the PD-L1 status in GC/GEJC neoadjuvant immunochemotherapy; further large-scale studies are needed.

Several studies have illustrated that the addition of radiotherapy (28, 30) or anti-VEGF drugs (20, 29) improved the effectiveness of neoadjuvant therapy. Our study reported that, compared with the addition of radiotherapy or anti-VEGF drugs with single neoadjuvant immunochemotherapy, the combination with radiotherapy did improve MPR rate but had a higher incidence of TRAEs. We even found that the combination of neoadjuvant immunochemotherapy with apatinib had worse MPR than neoadjuvant immunochemotherapy only. However, the results were relatively not stable only in the two studies for the addition of neoadjuvant radiotherapy and anti-VEGF drugs.

There are several limitations to this meta-analysis. First, some included studies are with conference abstract without available full text. Second, not every clinical trial reported all outcomes, and some have not yet reached their endpoint, which may affect the stability of the results. Third, the prognostic endpoints, like OS and DFS, were not included in this meta-analysis, because most studies are ongoing trials. Furthermore, most studies are single-arm clinical trials and those compared neoadjuvant immunochemotherapy with NACT are limited. Thus, large-sample, multicenter, and RCT studies are needed to evaluate these results. The variations in NACT regimens, study design, clinical parameters, and MMR status all contribute to heterogeneity.

## 5 Conclusions

This systematic review and meta-analysis found that PD-1/PD-L1 inhibitors combined with NACT for locally advanced GC/GEJC were well tolerated and may confer therapeutic advantages. The integration of ICIs into NACT has shown the potential for application in any PD-L1 expression population.

## Data availability statement

The datasets presented in this study can be found in online repositories. The names of the repository/repositories and accession number(s) can be found in the article/**Supplementary Material**.

## Author contributions

Concept and Design: BW, ZY, HC, WL, and SW. Collection and assembly of data: BC, WL, and LS. Data analysis and interpretation: GL, JH, LC, ZY, HC, and SW. Manuscript writing: ZY, HC, and SW. Manuscript revision: ZY, HC, and SW. All authors contributed to the article and approved the submitted version.
