# Supplementary material for: Combining neoadjuvant chemotherapy with PD-1/PD-L1 inhibitors for locally advanced, resectable gastric or gastroesophageal junction adenocarcinoma: A systematic review and meta-analysis
Source: Front Oncol. 2023 Jan 26;13:1103320. doi: 10.3389/fonc.2023.1103320 (PMC9909552; doi:10.3389/fonc.2023.1103320)
Supplement: Supplementary file 2 [file DataSheet_2.pdf]

Supplementary Materials

Supplementary Figure 1. The risk of bias of the included studies. (A) Risk of bias graph; (B) Risk of bias summary.

Supplementary Figure 2. Funnel plots of enrolled studies reported with (A) pCR outcome, (B) MPR outcome, (C) R0 resection rate outcome, (D) TRAEs outcome and (E) grade 3 to 4 TRAEs outcome.

Supplementary Figure 3. Sensitivity analysis for (A) MPR outcome, (B) R0 resection rate outcome, (C) TRAEs outcome and (D) grade 3 to 4 TRAEs outcome.

Supplementary Figure 4. Subgroup analysis based on ICI type for (A) R0 resection rate outcome, (B) TRAEs outcome and (C) grade 3 to 4 TRAEs outcome.

Supplementary Figure 5. Subgroup analysis based on treatment mode for (A) pCR outcome, (B) MPR outcome, (C) R0 resection rate outcome, (D) TRAEs outcome and (E) grade 3 to 4 TRAEs outcome.

Supplementary Figure 6. Subgroup analysis based on neoadjuvant chemotherapy regimen for (A) pCR outcome, (B) MPR outcome, (C) R0 resection rate outcome, (D) TRAEs outcome and (E) grade 3 to 4 TRAEs outcome.

Supplementary Table 1. Studies reported outcomes that neoadjuvant chemotherapy combined with immune checkpoint inhibitors comparing with single neoadjuvant chemotherapy in gastric adenocarcinoma.

Supplementary Table 2. Ongoing neoadjuvant chemotherapy combined with immune checkpoint inhibitors comparing with single neoadjuvant chemotherapy clinical trials in gastric adenocarcinoma.

Supplementary Table 3. Different combination of neoadjuvant chemotherapy and immunotherapy result in different grade 3-4 treatment-related adverse events.

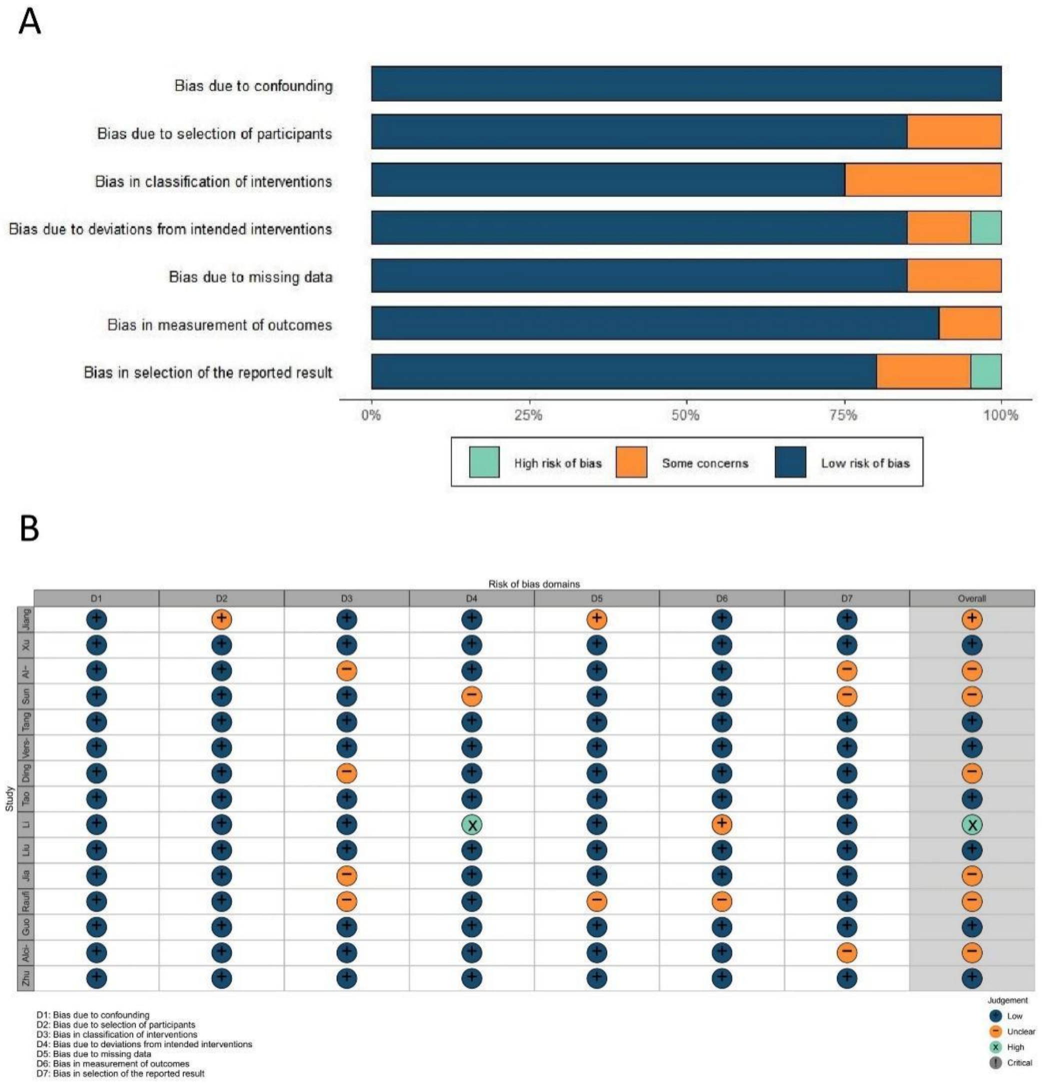

Supplementary Figure 1. The risk of bias of the included studies. (A) Risk of bias graph; (B) Risk of bias summary.

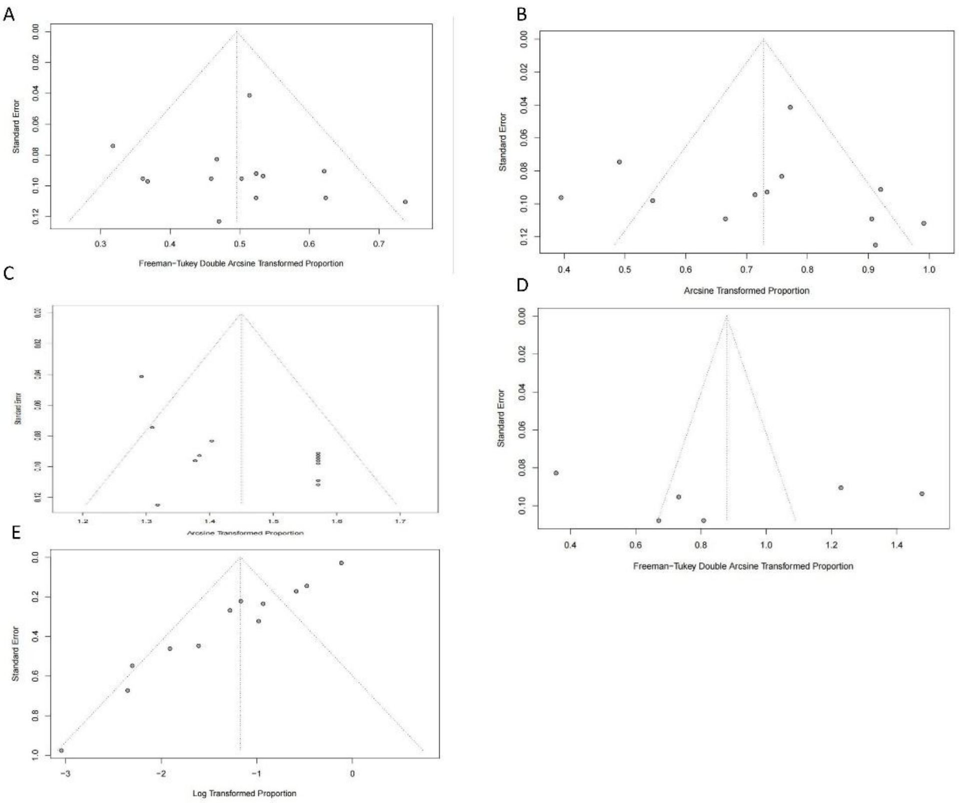

Supplementary Figure 2. Funnel plots of enrolled studies reported with (A) pCR outcome, (B) MPR outcome, (C) R0 resection rate outcome, (D) TRAEs outcome and (E) grade 3 to 4 TRAEs outcome.

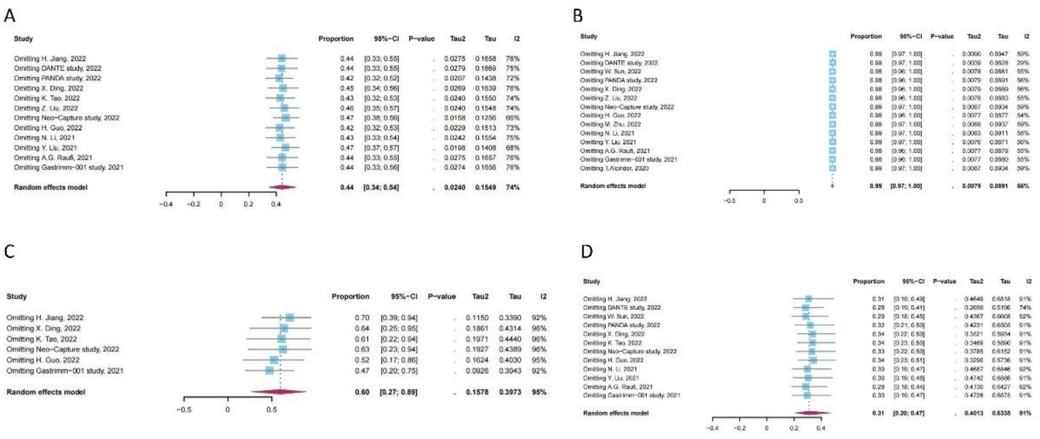

Supplementary Figure 3. Sensitivity analysis for (A) MPR outcome, (B) R0 resection rate outcome, (C) TRAEs outcome and (D) grade 3 to 4 TRAEs outcome.

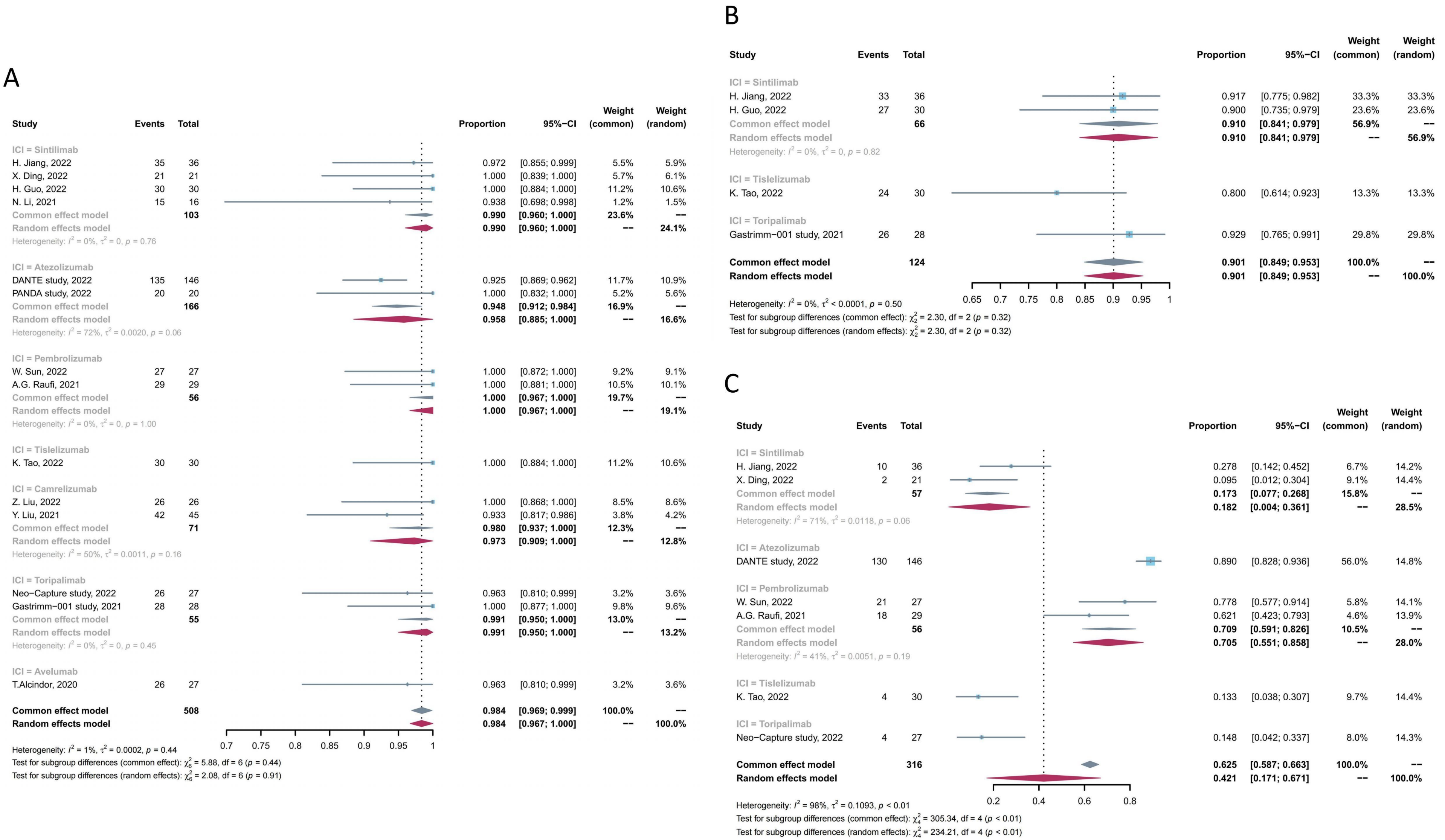

Supplementary Figure 4. Subgroup analysis based on ICI type for (A) R0 resection rate outcome, (B) TRAEs outcome and (C) grade 3 to 4 TRAEs outcome.

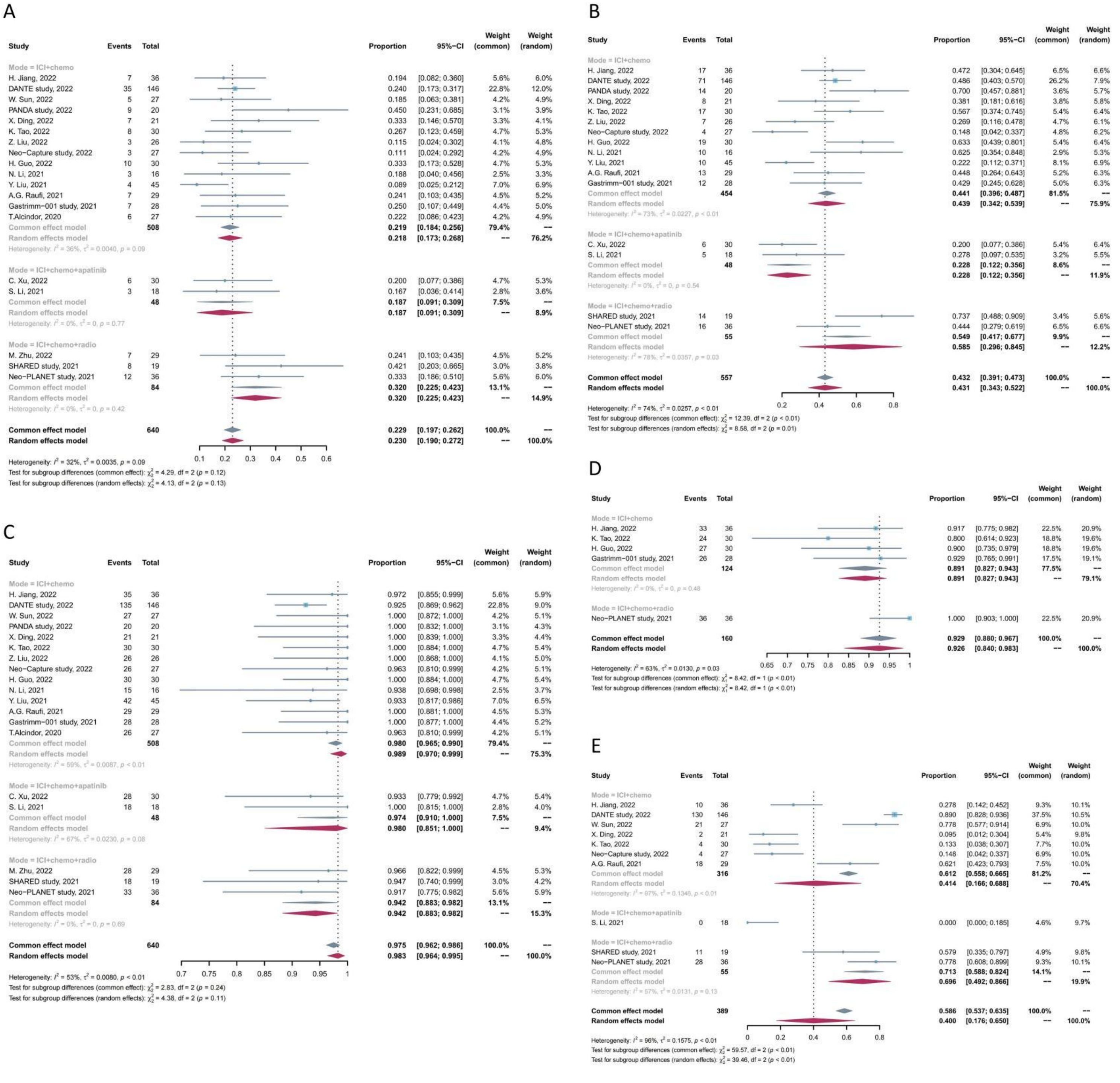

Supplementary Figure 5. Subgroup analysis based on treatment mode for (A) pCR outcome, (B) MPR outcome, (C) R0 resection rate outcome, (D) TRAEs outcome and (E) grade 3 to 4 TRAEs outcome.

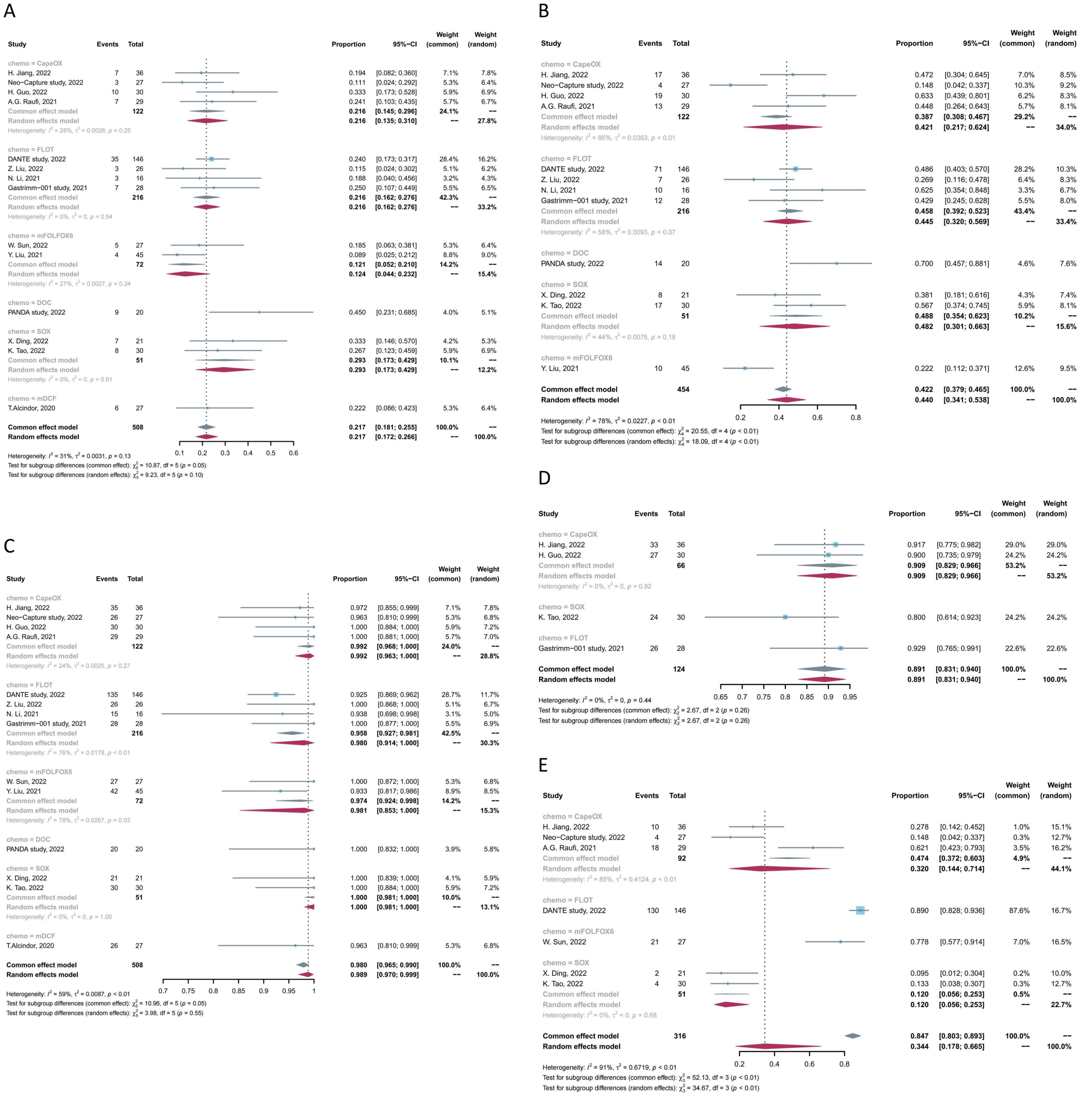

Supplementary Figure 6. Subgroup analysis based on neoadjuvant chemotherapy regimen for (A) pCR outcome, (B) MPR outcome, (C) R0 resection rate outcome, (D) TRAEs outcome and (E) grade 3 to 4 TRAEs outcome.

Supplementary Table1. Studies reported outcomes that neoadjuvant chemotherapy combined with immune checkpoint inhibitors comparing with single neoadjuvant chemotherapy in gastric adenocarcinoma.

Study	Year	Clinical trial identification	Study design	Group	No. of patients	R0 resection rate (%)	pCR rete (%)	Post operative pathologic stage
DANTE	2022	NCT03421288	multicenter, phase IIb trial.	A: FLOT+ atezolizumab<br>B: FLOT	A: 146; B:149	A: 92% vs.<br>B: 91%	-	A vs. B:<br>ypT0, 23% vs. 15%;<br>ypN0, 68% vs. 54%
Z. Liu	2022	ChiCTR2000030610	single-center, randomized, controlled clinical study	A: FLOT+ camrelizumab<br>B: FLOT	A: 26; B:21	A: 100% vs.<br>B: 90.5%	A: 11.5% vs.<br>B: 4.8%	A vs. B:<br>ypT0, 15% vs. 5%;<br>ypN0, 46% vs. 24%
J. Lin	2022	-	retrospective study	A: camrelizumab + S-1 + nab-paclitaxel<br>B: S-1 + nab-paclitaxel	A: 95; B: 33	-	A: 21.2% vs.<br>B: 5.3%	A vs. B:<br>ypT0, 24.2% vs 6.3%;<br>ypN0, 66.7%% vs. 38.9%%

Supplementary Table2. Ongoing neoadjuvant chemotherapy combined with immune checkpoint inhibitors comparing with single neoadjuvant Chemotherapy clinical trials in gastric adenocarcinoma.

Study	Status	First posted	Clinical trial identification	Study design	Experimental	Active comparator	No. of patients
A Pilot Study of Neoadjuvant Chemotherapy With or Without Camrelizumab for Locally Advanced Gastric Cancer	Recruiting	1-Nov-21	NCT05101616	Phase II	camrelizumab + paclitaxel +SOX	paclitaxel +SOX	100
Neoadjuvant Immunotherapy and Chemotherapy for Locally Advanced Esophagogastric Junction and Gastric Cancer Trial (NICE)	Recruiting	9-Feb-21	NCT04744649	Phase II	JS001+XELOX or SOX	XELOX or SOX	110
Study of Pembrolizumab (MK-3475) Plus Chemotherapy Versus Placebo Plus Chemotherapy in Participants With Gastric or Gastroesophageal Junction (GEJ) Adenocarcinoma (MK-3475-585/KEYNOTE-585)	Active, not recruiting	9-Oct-17	NCT03221426	Phase III	Pembrolizumab+XP or FP	XP or FP	1007
Study of Pembrolizumab (MK-3475) Plus Chemotherapy Versus Placebo Plus Chemotherapy in Participants With Gastric or Gastroesophageal Junction (GEJ) Adenocarcinoma (MK-3475-585/KEYNOTE-585)-China Extension	Recruiting	11-May-21	NCT04882241	Phase III	Pembrolizumab+XP or FP	XP or FP	120
A Clinical Study of HLX10 Combined With Chemotherapy Versus Placebo Combined With Chemotherapy for Neoadjuvant/Adjuvant Treatment of Gastric Cancer	Recruiting	25-Oct-19	NCT04139135	Phase III	HLX10+SOX	SOX	642

Supplementary Table3. Different combination of neoadjuvant chemotherapy and immunotherapy result in different grade 3-4 treatment-related adverse events.

Study<br>NACT<br>ICI	H. Jiang<br>CapeOx<br>Sintilimab	X. Ding<br>SOX<br>Sintilimab	K. Tao<br>SOX<br>Tislelizumab	Neo-Capture study<br>CapeOx<br>Toripalimab
Total grade 3-4 TRAEs	27.80%	9.50%	13.30%	14.80%
Anemia	13.90%	9.50%	3.10%	-
White blood cell count decrease	2.80%	-	-	-
Lymphocyte count decreased	-	-	-	-
Neutrophil count decrease	13.90%	-	6.30%	14.80%
Aspartate aminotransferase increase	2.80%	-	-	-
Platelet count decrease	2.80%	-	-	-
Platelet count decrease	2.80%	-	-	-
Myelosuppression	-	-	6.30%	-
Leukopenia	-	-	6.30%	-
Platelet count decrease	-	-	3.10%	-
Dermatitis	-	-	3.10%	-
Abdominal aortic thrombosis	-	-	3.10%	-
Nausea	-	-	-	-

TRAEs, treatment-related adverse events; ICI, immune checkpoint inhibitor; NACT, neoadjuvant chemotherapy.
