# Supplementary material for: Combining neoadjuvant chemotherapy with PD-1/PD-L1 inhibitors for locally advanced, resectable gastric or gastroesophageal junction adenocarcinoma: A systematic review and meta-analysis
Source: Front Oncol. 2023 Jan 26;13:1103320. doi: 10.3389/fonc.2023.1103320 (PMC9909552; doi:10.3389/fonc.2023.1103320)
Supplement: Supplementary file 3 [file Table_1.docx]

Dear Editor and reviewer，

Raw data and R code for this meta-analysis have been uploaded to Jianguoyun. Please access the data via the link below:

<https://www.jianguoyun.com/p/DcATzmgQ7ZiXCxiGiucEIAA>

Thank you for your valuable time for processing this manuscript.

Sincerely,

Bo Wei

Chinese PLA General Hospital
